# Post-traumatic Stress Disorder in Victims of Sexual Assault With Pre-assault Substance Consumption: A Systematic Review

**DOI:** 10.3389/fpsyt.2019.00092

**Published:** 2019-03-13

**Authors:** An Tong Gong, Sunjeev K. Kamboj, Helen Valerie Curran

**Affiliations:** Clinical Psychopharmacology Unit, Research Department of Clinical, Educational and Health Psychology, University College London, London, United Kingdom

**Keywords:** PTSD, sexual assault, substance use, intoxication, alcohol, self-blame, pre-assault alcohol use, social reaction

## Abstract

**Background:** Post-traumatic stress disorder (PTSD) and substance consumption commonly co-occur in victims of sexual assault. Substance consumption can occur pre- andi/or post-assault. Pre-assault substance consumption may have an impact on the subsequent development of PTSD. This review aims to provide an overview of current understanding of the effects of acute substance intoxication and chronic pre-assault problematic substance use on symptoms of PTSD amongst individuals who were victims of sexual assault.

**Methods:** PsycINFO, EMBASE, and MEDLINE were searched using terms related to PTSD, sexual assault, and substance consumption. These yielded 2,121 articles, 268 of which were retrieved for more detailed evaluation and 13 of these met inclusion criteria and were appraised in full.

**Results:** Overall, the reviewed papers supported our hypothesis that acute substance intoxication and chronic pre-assault problematic substance use are associated with fewer initial PTSD symptoms but less improvement over time, resulting in slower overall PTSD recovery. They also highlighted post-assault characterological self-blame and negative social reactions as mediators of recovery in the context of pre-assault substance consumption.

**Conclusions:** Acute substance intoxication and chronic pre-assault problematic substance use appear to have an impact on the development of PTSD symptoms amongst victims of sexual assault. The importance of developing early interventions and routine screening and assessment for PTSD and pre-assault substance consumption is emphasized. The limited research on male victims and on substances other than alcohol is highlighted.

## Introduction

### Rationale

#### Post-traumatic Stress Disorder (PTSD)

Over 70% of the general population report experiencing or witnessing a traumatic event that would qualify under criterion A1 of the diagnostic criteria for post-traumatic stress disorder [PTSD; Diagnostic and Statistical Manual of Mental Disorders: DSM-5; (1)]. In psychiatry and clinical psychology, traumatic events involve “exposure to actual or threatened death, serious injury, or sexual violence” (1), including interpersonal violence, road traffic accidents, and exposure to aversive details of trauma through electronic and online media. A proportion of people who experience traumatic events go on to develop PTSD, with a lifetime population prevalence of 2–15% (2).

PTSD symptoms include re-experiencing via intrusive memories, flashbacks and nightmares; (hyper)arousal in the form of exaggerated startle response and hypervigilance; and protective reactions, including emotional numbing, avoidance, amnesia and cognitive avoidance [DSM-5; (1)]. In addition, PTSD commonly presents with negative cognitions and affect, including anger, sadness and guilt. The severity and course of PTSD symptoms vary across individuals.

#### Substance Consumption and Substance Use Disorders (SUDs)

The use of substances may involve licit (alcohol and cigarettes, and cannabis in some jurisdictions) and/or illicit substances. Substance consumption is an important public health issue with substantial morbidity and social/economic costs (3).

Frequent and excessive use of substances may result in the development of substance use disorders (SUDs) in vulnerable individuals. Individuals with SUDs show impaired control over their use of substances. They may experience cravings and use the substance in larger amounts or over a longer period despite a persistent desire to regulate or discontinue use. Furthermore, SUDs are usually accompanied by social impairment, risky use of substances and symptoms of tolerance and withdrawal [DSM-5; (1)]. In the United Kingdom, the prevalences of drug use were approximately 8.4% in adults and 10% in children, respectively (4). In the United States, nationally representative surveys showed that 12-month and lifetime prevalences of SUDs were 3.9 and 9.9%, respectively (5), alcohol use disorders (AUDs) 13.9 and 29.1% (6), and nicotine use disorders (NUDs) 20.0 and 27.9% (7).

SUDs occur in a broad range of severity, from mild to severe, with severity based on the number of symptom criteria endorsed. Generally, a mild SUD is suggested by the presence of two to three symptoms, moderate by four to five symptoms, and severe by six or more symptoms [DSM-5, (1)]. Therefore, for the sake of consistency, the term “SUDs” will be used throughout this current review wherever it is evident that publications were referring to a pattern of substance consumption that is consistent with the definition of SUDs as provided in DSM-5. Where there is less certainty about the degree of substance use *disorder*, the term “problematic substance use” is used. This can refer to a *pattern* of ongoing problematic use. Given that non-substance-related disorders (i.e., behavioral addictions) such as gambling disorders [DSM-5, (1)] are not primarily associated with the vulnerability engendered by SUDs (e.g., impaired memory, judgement, and consciousness), these are not considered in the current review.

#### The Relationship Between PTSD and SUDs

PTSD and SUDs commonly co-occur (5). PTSD is comorbid with the use of various types of drugs, for example, heroin, cocaine, and amphetamines, and most commonly with alcohol and nicotine use (8–10). Those with PTSD were 2–4 times more likely than those without PTSD to meet criteria for SUDs. Among individuals with PTSD, nearly half (46.4%) also met criteria for SUDs. In addition, patients with concurrent PTSD and SUDs show higher symptom severity and poorer treatment outcomes compared to patients with either disorder alone (11). However, beyond prevalence of comorbid PTSD-SUD, the relationship between these disorders is complex and bidirectional. Extensive research has focused on delineating this relationship. A number of theoretical accounts of the relationship between PTSD and SUDs have been posited, and these will briefly be reviewed below.

##### Self-medication model

The self-medication model proposes that trauma survivors' excessive use of substance is an attempt to alleviate PTSD symptoms (12). This model suggests that the use of substances is maintained through negative reinforcement, for example, via temporary relief from negative affect and other aversive symptoms associated with trauma. In other words, the *function* of substance use is to (down-) regulate emotional pain resulting from trauma in order to reestablish “emotional homeostasis.” This model posits a degree of psychopharmacological specificity in that the substance used to regulate emotions is expected to psychophysiologically alleviate aversive affects that vary from individual to individual. For example, cocaine may be used to regulate low energy and depression, nicotine to remedy dysphoria, and alcohol to relieve anxiety (13, 14). In PTSD, furthermore, corticotropin-releasing hormone and noradrenergic systems may interact such that the stress response is progressively augmented. Patients may use a range of substances in an effort to interrupt this progressive augmentation (15). Evidence from epidemiological and longitudinal studies provides some support for the self-medication model, such that young adults with early-life trauma tend to use drugs to self-medicate troubling trauma-associated memories, nightmares, or painful hyperarousal symptoms (16).

##### Negative reinforcement model

Although the self-medication hypothesis posits a central role for negative reinforcement, the function of substance use is to regulate symptoms of PTSD. In contrast, the negative reinforcement model—a more general theory of problematic substance consumption—suggests that drug withdrawal-driven negative affect is the fundamental motivator for the use of substances and that PTSD may lead to a greater sensitivity to such effects, hence indirectly increasing an individual's potential to develop and maintain ongoing problematic substance consumption (17). In contrast to the self-medication model, this model makes no prediction about psychopharmacological specificity between the substance of choice and the specific state of psychological distress an individual with PTSD might be experiencing. Evidence from several laboratory-based studies supports this model [for review, see (18)], highlighting the role of withdrawal symptoms (19) and a lack of substance and affective specificity (20).

##### Mutual maintenance model

An extension of the self-medication model—the mutual maintenance model—posits a reflexive relationship between SUDs and PTSD symptoms (21, 22). This model suggests that repeated use of substances not only helps *temporarily* suppresses PTSD symptoms, but may simultaneously impede natural recovery from PTSD in the *longer term*. For example, while exposure to reminders of the traumatic experience, such as similar people, situations, or places, can trigger substance consumption (17), withdrawal symptoms from substances, such as palpitations, sweating, and shivering, are similar to fear responses during the traumatic event and can evoke traumatic memories that trigger PTSD symptoms (23), which may in turn be exacerbated and maintained over time. Neuroendocrine research also provides some evidence for this model, as the acute and chronic stress in PTSD negatively affects hippocampal function, which can be further impaired by chronic alcohol exposure, and especially alcohol withdrawal (24).

##### High-risk and susceptibility hypotheses

The high-risk and susceptibility hypotheses propose alternative pathways linking PTSD and SUDs (25, 26). The high-risk hypothesis suggests that engaging in substance consumption and related “high-risk” activities (e.g., being intoxicated in dangerous situations) increases the probability of experiencing a traumatic event, and hence of developing PTSD. The susceptibility hypothesis suggests that excessive use of substances may play a causal role, in that substance users may be more susceptible to PTSD following a traumatic event due to impaired psychological processes (e.g., judgement, inhibition, decision-making, and memory) and/or primed neurochemical systems resulting from extensive substance consumption. A number of studies have demonstrated that excessive substance consumption contributes to rape vulnerability and increases susceptibility to the development of PTSD (27, 28). In addition, multiple studies with female substance abusers also demonstrate high rates of revictimization in the form of intimate partner violence, as well as stranger rape and physical assault in adulthood, and subsequent development of PTSD (27, 29, 30).

##### Third variable model

The third variable model postulates that concurrent PTSD and SUDs may be due to an unknown shared third variable, involving as-yet unspecified biological vulnerability and/or personality factors (31–34). In addition, several studies suggest that the relationship between PTSD and SUDs may be mediated by other factors, such as poor coping skills, self-regulatory deficits, and trauma-related cognitions (35, 36). For example, high anxiety sensitivity appears to partially mediate the relationship between PTSD and SUDs (37).

#### The Relationship Between PTSD and SUDs in Interpersonal Violence

Interpersonal violence refers to violence between individuals, including within families and between acquaintances and strangers (38). Interpersonal violence is further differentiated into sexual and non-sexual assault. Non-sexual assault takes place when an individual or a group provokes and attacks a person physically without overt sexual contact. Non-sexual assault includes physical assault (i.e., physical attacks with or without the use of a weapon), threats or menacing and unwanted contact, such as shoving, pushing, tripping, without necessarily resulting in physical harm. In this review, the term “sexual assault” refers to an act in which a person sexually touches, coerces or physically forces a person to engage in a sexual act against their will. This broad category of sexual violence includes rape (forced vaginal, anal or oral penetration or drug-facilitated sexual assault), groping, child sexual abuse, sexual torturing, and sexual harassment (38). Physical assault and sexual assault may also co-occur in certain situations (39).

Many studies have demonstrated the co-occurrence of PTSD and SUDs in victims of interpersonal violence, including both sexual and non-sexual assaults, and focused on investigating their temporal relationship. Generally, these studies describe a complex temporal relationship between PTSD and SUDs in the context of interpersonal violence [e.g., (40)]. Substance consumption can occur pre- and post-assault and may result in peri-assault intoxication. The types of relationship are broadly summarized in the following three categories: acute substance intoxication, chronic pre-assault problematic substance use and post-assault SUDs.

##### The effect of acute substance intoxication on PTSD

Acute substance use immediately or shortly before an episode of interpersonal violence involves the victims' consumption, either voluntarily or involuntarily, of psychoactive substances, which can lead to various levels of intoxication and/or incapacitation prior to and during the incident (i.e., pre- and peri-assault intoxication). Overall, the evidence for a role of acute substance intoxication in PTSD has been inconsistent. Some studies have suggested an increased risk of PTSD, with a more chronic and severe course of symptoms in victims of interpersonal violence with acute intoxication (41–43), whereas others indicate a protective effect of acute intoxication (22). In addition, a number of studies have demonstrated that acutely intoxicated sexual assault victims may further develop chronic substance use problems comorbid with PTSD and depression after an assault (44).

##### The effect of chronic pre-assault problematic substance use on PTSD

The effect of chronic pre-assault problematic substance use on PTSD has also been investigated. This includes those whose SUD symptoms (e.g., negative consequences such as hangover and loss of interest in activities and hobbies) predated the occurrence of interpersonal violence by at least a month. It should be noted that victims with chronic pre-assault problematic substance use patterns may have been either intoxicated or sober during the index incident of interpersonal violence. Similar to studies investigating the effect of acute intoxication on PTSD, findings on those with pre-existing substance use problems are mixed, with some studies suggesting a relationship (45), while others have failed to find any relationship (46, 47).

##### The relationship between PTSD and post-assault SUDs

In addition, a number of studies have been conducted to investigate the opposite relationship, implying that SUD develops as a consequence of PTSD resulting from interpersonal violence. These studies control for the effects of pre-existing chronic problematic substance use or acute intoxication prior to and/or during the assault incident resulting in PTSD by only recruiting participants without such a history. Evidence regarding the onset and development of SUDs after assaults has been mixed. Some studies have shown that neither trauma exposure nor the presence of PTSD significantly predicts the onset of SUDs (48, 49), while others demonstrated a greater likelihood of subsequent development of SUDs in people meeting criteria for PTSD, supporting the self-medication model (50, 51) and the negative reinforcement model (52, 53).

### Objectives

In summary, the relationship between PTSD and SUDs in interpersonal violence appears complex and inconsistent. A number of authors have suggested models to account for the relationship between PTSD and SUDs, but there have been no systematic reviews of this relationship in the context of interpersonal violence. The current review will primarily focus on the effect of acute substance intoxication and chronic pre-assault problematic substance use on the development of PTSD symptoms, specifically amongst victims of sexual assault occurring in adolescence and adulthood. In other words, we focus on studies in which chronic problematic pre-assault substance use or acute substance intoxication has temporal precedence (and hence may be causally implicated) relative to PTSD onset. This contrast with the substantial existing research and reviews on the relationship between PTSD and the development of *post-assault* SUDs [e.g., (54, 55)]. In addition, in the extant relevant research, the sample population, the type of substances and the type of sexual assaults differ, which in turn may contribute to mixed results. Several past reviews have reported the specific effects of acute alcohol intoxication and chronic pre-assault problematic alcohol use on PTSD symptoms [e.g., (56)]. In contrast, this current review will be broader and cover various types of substances to systematically integrate data from these studies to determine if any systematic pattern of results emerges.

### Research Questions

We focus primarily on two questions:

1. What are the effects of acute substance intoxication and chronic pre-assault problematic substance use on the development of PTSD symptoms amongst victims of sexual assault?
2. Which mediators have been investigated that might modulate the effects of acute substance intoxication and chronic pre-assault problematic substance use on PTSD symptoms amongst victims of sexual assault?

## Methods

### Systematic Review Protocol

The Preferred Reporting Items for Systematic Reviews and Meta-analyses statement (PRISMA) (57) was used to guide the protocol design and as a basis for reviewing and reporting on relevant papers.

### Study Designs, Participants, Interventions, Comparators, and Outcomes

Studies that investigate the effect of various licit and illicit substances and sexual assault that occur in the presence or absence of physical assault were included. In contrast to the majority of past studies and reviews that have examined female victims only [e.g., (56, 58, 59)], the current review will include studies of both female and male adult and adolescent (≥14 years old) victims of sexual assault, given the growing recognition of the effects of sexual assault in males. Acute substance intoxication and chronic pre-assault problematic substance use are limited in victims of childhood sexual assault. Because of this, only sexual assault that occurred in adolescence and adulthood is included here. As this review examines assault experiences and pre-assault substance consumption retrospectively in clinical populations, we include both longitudinal and cross-sectional studies, which compare PTSD symptoms in assault victims with and without pre-assault substance consumption.

### Search Strategy and Data Sources

A systematic literature search was carried out using three electronic databases (PsycINFO, EMBASE, and MEDLINE). Search terms related to PTSD were combined with terms associated with substance consumption and sexual assault. The search terms selected were intentionally inclusive and included multiple synonyms in order to ensure that studies considering a wide range of outcomes would be identified. The databases were searched for articles published on or before 11th August 2018.

### Studies Sections and Data Extraction

The first author performed the initial data extraction by removing duplicates and all the articles that appeared clearly irrelevant on the basis of the relevance of the title and after reading the specific abstract. The full-text of the remaining studies were independently assessed for eligibility by two researchers. After a full-text evaluation of the potentially relevant studies, the two researchers reached a consensus regarding eligibility and excluded all the research articles that did not meet the inclusion criteria.

### Data Analysis

In view of the highly heterogeneous study characteristics, a meta-analysis/meta-regression was not considered appropriate to represent the richness of study data and findings. On this basis, for each of the included studies, we provided a detailed synthesis of relevant data based on the objectives of this review, by reporting in detail the qualitative effects of acute substance intoxication and chronic pre-assault problematic substance use, respectively, on the severity and course of post-assault PTSD symptoms, and significant mediators (if any). Other relevant data, such as the characteristics of participants, substance and assault types and the use of different measures were noted and summarized for each study in relevant tables.

### Critical Appraisal of Articles

The Newcastle-Ottawa Scale [NOS; (60)] was used to assess quality of studies in this review. The NOS offers a star rating system modified for cohort/longitudinal and cross-sectional studies, respectively, specific to this review (see Supplementary Materials). Using the NOS, each study is judged on multiple items, categorized into three groups. Firstly, “selection” items refer to the representativeness and selection of the study groups and the ascertainment of experimental groups. Secondly, “comparability” items examine the comparability of the study groups on the basis of design and/or analysis. Thirdly, “outcome” items assess the quality of outcomes. The overall rating system of quality for the current review was developed based on NOS “star system.” The studies judged to have the highest quality were awarded up to 10 stars for cohort/longitudinal studies and eight stars for cross-sectional studies. Studies earning seven or more stars were rated as “high” in both relevance and quality, studies scoring five or six were rated as “medium,” and studies scoring <5 were rated as “low.” The quality of studies was evaluated by two authors independently. When there was a discrepancy in rating, the authors discussed their ratings before reaching a consensus for each included study.

## Results

### Inclusion Criteria

Studies meeting the following inclusion criteria were included: (a) the effect of substance consumption was being investigated; (b) the study reported sexual assault in adolescence and adulthood (i.e., age 14 years or older); (c) the study included measures of PTSD symptoms; (d) the study assessed acute substance intoxication and chronic problematic substance use prior to and during sexual assault; (e) the study was published in a peer-reviewed journal; (f) the study was published in English; and (g) the study was published after January 2000, as Ullman's (59) review on the link between substance consumption and adult sexual assault covered most relevant studies prior to this date. Studies meeting these criteria were subjected to formal quality and relevance assessment.

### Study Selection

**Figure 1:**
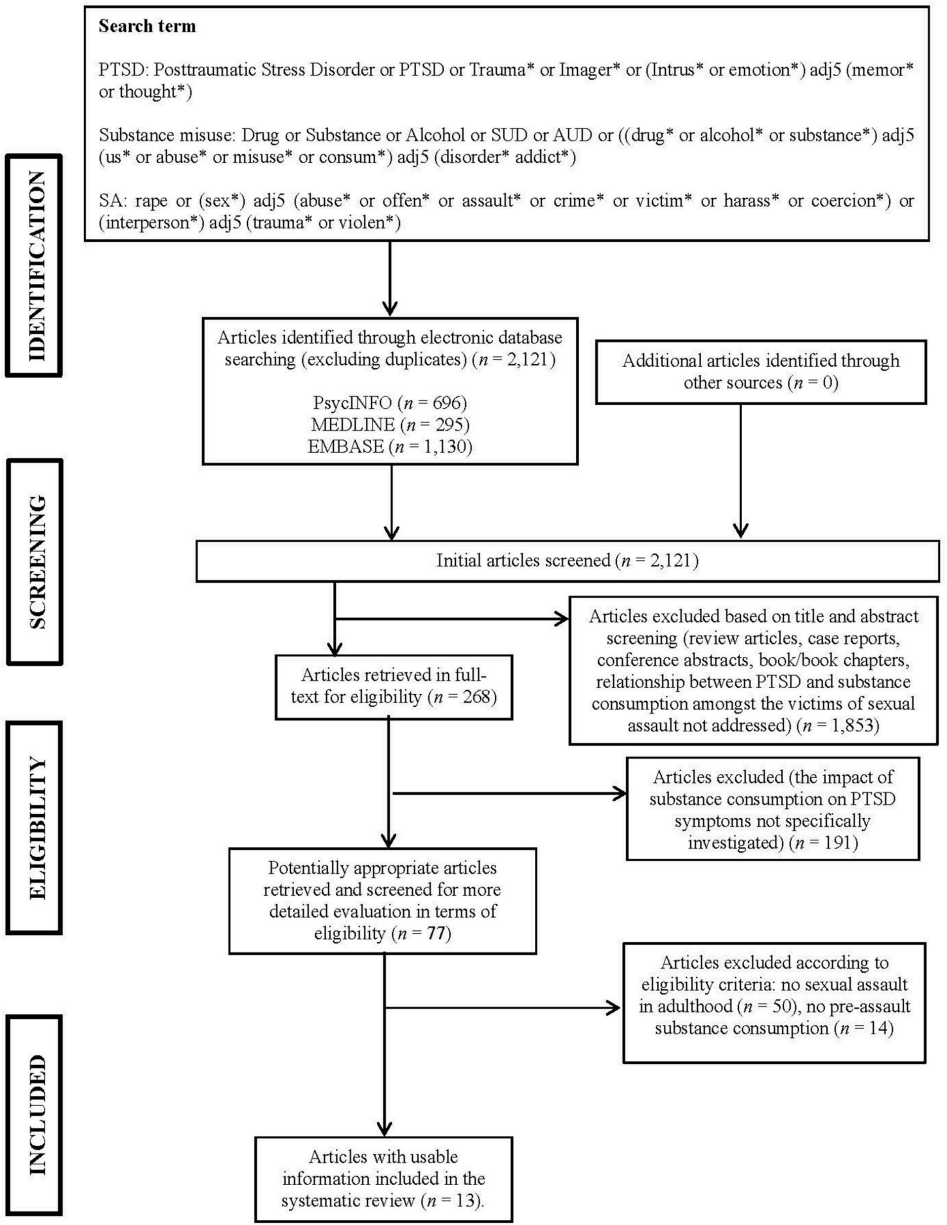
PRISMA flowchart of the systematic search.

Once duplicates were removed, the database search yielded 2,121 unique studies. Titles and abstracts of these studies were retrieved for more detailed evaluation. In the first round of selection, if an abstract appeared to represent a relevant article (excluding review articles, case reports, conference abstracts, books/book chapters) considering the relationship between substance consumption and PTSD, the full article was read to determine if the study met the inclusion criteria (*n* = 268). In the second round, 191 of the 268 references were excluded because they did not address the impact of substance consumption on PTSD but instead focused on other aspects of the relationship (e.g., treatment for PTSD and SUDs; the prevalence of co-occurrence). Of the remaining 77 articles, 50 articles were further excluded because they focused on the development of SUDs as a result of childhood sexual abuse. In the last round, 14 articles were excluded for lack of clarity as to whether they considered pre- or post-assault substance consumption. As a result, a total of 13 studies remained that met the inclusion criteria. Figure 1 illustrates the selection process for the relevant articles.

### Risk of Bias

Quality and relevant ratings for the 13 articles included in this review are summarized in Table 1. As can be seen from the table, all studies were judged to be of medium to high quality. In general, the “comparability” item of the appraisal tool was consistently scored low. Specifically, the major shortcomings of reviewed studies were the biases associated with participants' gender representation, the assessment of substance exposure and its effects on PTSD symptoms, the lack of study control on baseline participant characteristics, and (short) length of follow-up assessments (in longitudinal studies).

**Table 1:** Quality and relevance ratings.

**Study**	**Year**	**Selection**	**Comparability**	**Outcome**	**Overall rating**
**LONGITUDINAL/COHORT STUDIES**					
Kaysen et al. (45)	2006	^***^	^*^	^*^	Medium
Kaysen et al. (41)	2010	^***^	^*^	^***^	High
Kaysen et al. (21)	2011	^***^	^*^	^***^	High
Peter-Hagene and Ullman (61)	2015	^***^	^*^	^**^	Medium
Blayney et al. (62)	2016	^***^	^*^	^**^	Medium
Peter-Hagene and Ullman (63)	2018	^***^	^*^	^*^	Medium
**CROSS-SECTIONAL STUDIES**					
Brown et al. (Study 1) (64)	2009	^***^	^*^	^*^	Medium
Brown et al. (Study 2) (64)	2009	^***^	^*^	^**^	Medium
Littleton et al. (65)	2009	^**^	^**^	^**^	Medium
Zinzow, Resnick, McCauley et al. (66)	2010	****	^*^	^**^	High
Zinzow, Resnick, Amstadter et al. (67)	2010	****	^*^	^**^	High
Zinzow et al. (68)	2012	****	^**^	^**^	High
Masters et al. (69)	2015	****	^**^	^**^	High
Jaffe et al. (70)	2017	^***^	^*^	^**^	Medium

* *NOS, Newcastle-Ottawa Scale. Star system that indicates that quality of each article, i.e., articles earning a higher number of stars are rated as higher in relevance and quality its higher quality*.

### Synthesized Findings

Tables 2A, B display basic details of the 13 articles retrieved by the search strategy described above. Twelve of these studies included only female victims, while one included both females and males (62). Eleven studies examined sexual assault victims, and two studies investigated victims of both sexual and physical assault (21, 41). The samples in these studies were wide ranging in terms of size (*n* = 64 to 3,001), setting (community, college, criminal justice system, hospital, health, and human services, and victims' service agencies), and socio-economic status of participants. All studies were conducted in the United States. Six studies were cross-sectional; seven were longitudinal. These studies utilized (semi-)structured and/or diagnostic interviews, surveys and questionnaires for data collection.

**Table 2A:** Details of longitudinal/cohort studies included in the current review.

**Study**	**Year**	***N***	**Population**	**Age (years) mean (range)**	**Gender**	**Substance type**	**Substance consumption**	**Assault type**	**PTSD measure**	**Substance consumption measure**	**Data analysis method**
Kaysen et al. (45)	2006	108	Criminal justice system, hospitals, victims' service agencies	31.48 (18–55)	F	Alcohol	Pre-assault AUDs	SA/PA^*^	CAPS^a^	SCID-NP-III-R-AUD^b^	MANOVA
Kaysen et al. (41)	2010	47	Community, hospitals, health, and human services	35.6 (19–53)	F	Alcohol/ drug	Alcohol/drug intoxication; peak alcohol use	SA/PA^*^	CAPS^a^	STI^a^; TLFB^b^	HLM
Kaysen et al. (21)	2011	64	Community, hospitals, victims' service agencies	35.6 (19–53)	F	Alcohol	Pre-assault AUDs; peak alcohol use; alcohol-related negative consequences	SA/PA^※^	CAPS^a^	TLFB^b^; SCID-IV-SUD^b^; DrInC-2R^c^	HLM
Peter-Hagene and Ullman, (61)	2015	877	Community	34.51 (18–69)	F	Alcohol	Alcohol intoxication	SA^※^	PDS^c^	MSES^c^	Cluster analysis
Blayney et al. (62)	2016	116	College students	23.04 (18–24)	19% M; 81% F	Alcohol	Drinking frequency; HED; alcohol-related negative consequences; alcohol intoxication	SA^*^[Table-fn TN5]	PCL-C^c^	R-SES^c^; DDQ^c^; YAACQ^c^	SEM
Peter-Hagene and Ullman, (63)	2018	1,013	Community	37.89 (18–71)	F	Alcohol	Alcohol intoxication	SA^※^	PDS^c^	R-SES^c^	HLM

*F, female; M, male; SA, sexual assault; PA, physical assault; PTSD, post-traumatic stress disorder; AUD, alcohol use disorders; CAPS, Clinician-Administered PTSD Scale (71); PDS, Post-traumatic Stress Diagnostic Scale (72); PCL-C, PTSD Checklist-Civilian Version (73); TLFB, Timeline Follow-Back Interview (74); SCID-IV-SUD, Structured Clinical Interview for DSM-IV, substance use disorder module (75); DrInC-2R, Drinker Inventory of Consequences (76); SCID-NP-III-R-AUD, Structured Clinical Interview for DSM-III-R Non-Patient Version, alcohol abuse, and dependence module (77); R-SES, revised Sexual Experiences Survey (78); HED, Heavy episodic drinking; DDQ, Daily Drinking Questionnaire (79); YAACQ, Young Adult Alcohol Consequences Questionnaire (80); MSES, Modified Sexual Experiences Survey (81); STI, Standardized Trauma Interview (82); HLM, hierarchical linear model (83); SEM, structural equation modeling (84); MANOVA, multivariate analysis of variance*.

a Diagnostic interview.

b (Semi-)structured interview.

c Surveys/questionnaires.

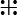
Assault experiences during the college years were assessed.

* Most recent rape experience in adulthood and adolescence was assessed. For individuals with multiple rapes, first incident of rape was assessed.

※ *The most distressing assault experience in adulthood and adolescence was assessed*.

**Table 2B:** Details of cross-sectional studies included in the current review.

**Study**	**Year**	***N***	**Population**	**Age (years) mean (range)**	**Gender**	**Substance type**	**Substance consumption**	**Assault type**	**PTSD measure**	**Substance consumption measure**	**Data analysis method**
Brown et al. (Study 1) (64)	2009	265	College students	19 (18–22)	F	Alcohol/drug	Alcohol/drug intoxication	SA^※^	PSS^b^	MSES^b^	MANOVA
Brown et al. (Study 2) (64)	2009	244	Community	24 (18–30)	F	Alcohol/drug	Alcohol/drug intoxication	SA^*^	NSW-PTSD^b^	MSES^b^	MANOVA
Littleton et al. (65)	2009	340	College students	21.6 (18–54)	F	Alcohol/drug	Alcohol/drug intoxication; pre-assault AUDs	SA^※^	PSS^b^	ACQ^b^; AUDIT^b^	ANOVA; linear regression
Zinzow, Resnick, McCauley et al. (66)	2010	2,000	College students	20.13	F	Alcohol/drug	Alcohol/drug intoxication	SA^*^	NSW-PTSD^a^	REI^a^	LRA
Zinzow, Resnick, Amstadter et al. (67)	2010	3,001	Community	46.58 (18–76)	F	Alcohol/drug	Alcohol/drug intoxication	SA^*^	NSW-PTSD^a^	REI^a^	LRA
Zinzow et al. (68)	2012	3,001	Community	46.58 (18–76)	F	Alcohol/drug	Alcohol/drug intoxication	SA^*^	NSW-PTSD^a^	REI^a^; NSW-AA/DA^a^	LRA
Masters et al. (69)	2015	667	Community	24.78 (21–30)	F	Alcohol/drug	Alcohol/drug intoxication; HED	SA^†^	TSI^b^	R-SES^b^; HED questionnaire^b^	LCA
Jaffe et al. (70)	2017	143	Community	22.00 (18–26)	F	Alcohol	Alcohol/drug intoxication; level of intoxication	SA^※^	PCL-C^b^	MSES^b^	NBHM; multivariate models

F, female; SA, sexual assault; PA, physical assault; PTSD, post-traumatic stress disorder; AUD, alcohol use disorders; TSI, Trauma Symptom Inventory (85); NSW-PTSD, National Women's Study PTSD module (86, 87); PSS, PTSD Symptom Scale (88); NSW-AA/DA, National Women's Study alcohol/drug abuse module (89, 90); PCL-C, PTSD Checklist-Civilian Version (73); REI, rape experience interview; TLFB, Timeline Follow-Back Interview (74); R-SES, revised Sexual Experiences Survey (78); HED, Heavy episodic drinking; MSES, Modified Sexual Experiences Survey (81); ACQ, Assault Characteristics Questionnaire (91); AUDIT, Alcohol Use Disorder Identification Test (92, 93); NBHM, negative binomial hurdle model (94); LRA, logistic regression analyses; MANOVA, multivariate analysis of variance; ANOVA, univariate analyses of variance; LCA, latent class analyses (95).

a (Semi-)structured interview.

b Surveys/questionnaires.

* Most recent rape experience in adulthood and adolescence was assessed. For individuals with multiple rapes, first incident of rape was assessed.

※ The most distressing assault experience in adulthood and adolescence was assessed.

† *Assault experiences in adolescence and adulthood (i.e., at age 14 years or older) were assessed*.

In the literature on pre-assault substance consumption, an important distinction is commonly made between acute substance intoxication and chronic pre-assault problematic substance use. In the current review, the presentation of the studies is therefore structured according to this distinction. Two studies assessed the effects of chronic pre-assault problematic substance use on PTSD (21, 45), while 11 studies investigated the effects of acute substance intoxication on the development of PTSD amongst victims of sexual assault.

The studies examining the effects of acute substance intoxication varied in their designs: four studies compared PTSD symptoms in assault victims with and without acute substance intoxication (41, 62, 63, 70); five studies categorized sexual assault experiences into types and compared their unique effects on PTSD symptoms (64–68); two studies identified subgroups of victims based on reported sexual assault characteristics and compared these subgroups with one another to investigate effects of these characteristics on PTSD symptoms (61, 69). Due to the advantages of longitudinal over cross-sectional studies in more effectively examining the effect of pre-assault substance consumption on the development, and especially the course, of PTSD symptoms, longitudinal studies are given more weight and hence are presented in advance of cross-sectional studies in this review. Similarly, more emphasis is placed on studies with larger sample size and higher quality and relevance ratings. In general, studies with higher quality and relevance ratings are presented in advance of studies with lower ratings.

Of the 13 studies, three studies investigated factors mediating the effect of acute substance intoxication on PTSD (61–63). All three studies are longitudinal, with medium quality and relevance ratings. All three examined acute alcohol intoxication and identified two mediators: self-blame and social reactions, which will be subsequently elaborated in this review.

#### Longitudinal Studies of Acute Substance Intoxication

Kaysen et al. (41) used independent samples and compared PTSD symptoms in 47 victims of sexual or physical assault who were intoxicated as a result of alcohol and/or drug consumption shortly before the assault with victims who were unintoxicated. The PTSD symptoms were assessed at three timepoints: 2–5 weeks post-assault, 3 months post-assault, and 6 months post-assault. After controlling for victims' perceived threat of the assault (i.e., subjective appraisal of risk and certainty of harm) and maximum number of drinks the victim consumed in the month prior to the first assessment, the authors found that assault victims who were non-intoxicated had significantly more initial intrusive symptoms at 2–5 weeks post-assault than did victims who were intoxicated. Over time, however, the non-intoxicated assault victims had a significantly steeper drop-off in intrusive symptoms, suggesting a quicker recovery and shorter course of PTSD symptoms following the assault. There were no significant differences between unintoxicated and intoxicated assault victims in avoidance and hyperarousal symptoms. As a methodological weakness, this study had a small sample size and did not report the number of physical or sexual assault victims, respectively, nor differentiate between them in their PTSD symptoms.

In a large-scale study, Peter-Hagene and Ullman (63) examined PTSD symptoms longitudinally in 1,013 victims of sexual assault who had and had not consumed alcohol shortly before the assault. Participants were assessed annually over the course of 3 years. Overall, the occurrence of PTSD symptoms declined over time, indicating recovery from the traumatic consequences of the assault in both groups. However, the intoxicated group had fewer PTSD symptoms initially, but there was no significant interaction effect between group (intoxicated and non-intoxicated) and time, suggesting that the differences in PTSD symptoms between intoxicated and non-intoxicated victims did not diminish over time. Therefore, the intoxicated group seemed to continue to display fewer PTSD symptoms over time.

Blayney et al. (62) examined both cumulative and most recent sexual assault experiences during university in 116 students. Cumulative sexual assault refers to the number of times an individual was exposed to sexual assault over the course of the 4 years at university. Participants reported their sexual assault experiences, levels of acute alcohol intoxication, PTSD symptoms and drinking behaviors (i.e., drinking frequency, number of binge drinking and alcohol-related negative consequences for the 30 days prior to the assessment) at the end of their 5th post-matriculation year (i.e., approximately 1 year after graduating from university). After 5 months, they were subsequently assessed on sexual assault re-victimization, and re-assessed on PTSD symptoms and drinking behaviors. In examining cumulative experiences of sexual assault, a proportion score (number of assaults during intoxication out of total number of assaults) was calculated to reflect the extent to which acute alcohol intoxication was potentially implicated as a risk factor for assault. In examining the most recent experience of sexual assault, the number of drinks and subjective rating of intoxication at the time of assault were recorded to represent the levels of acute alcohol intoxication. In terms of both cumulative and most recent experiences of sexual assault, the findings suggested that greater levels of acute alcohol intoxication predicted more problematic post-assault drinking behaviors, but not PTSD severity. However, after controlling for participants' drinking behaviors and PTSD severity at the end of their 5th post-matriculation year, the relationship between acute alcohol intoxication and post-assault drinking behaviors was no longer significant.

Peter-Hagene and Ullman (61), using a data-driven approach, identified subgroups of victims based on reported sexual assault characteristics and subsequently compared with these subgroups to investigate effects on PTSD symptoms. This study used cluster analysis to create composite variables that encompassed both alcohol and violence information. This study included assault characteristics identified by previous research to be most relevant to (poor) recovery from PTSD, including (1) victim and perpetrator's intoxication, (2) level of physical violence used by the perpetrator during the assault, (3) victims' negative peri-traumatic responses such that how much they thought their life was in danger and how upset they were at the time of the assault, and (4) the victim's relationship with the perpetrator. Three significantly different categories of sexual assault emerged from the data: (a) “alcohol-related assaults” (cluster encompassing alcohol-related assault and moderate levels of violence, fear and distress); (b) “high-violence assaults” (cluster with the most violent experiences and severe assaults); and (c) “moderate sexual-severity assaults” (cluster containing the lowest levels of sexual assault severity and physical violence). Peter-Hagene and Ullman (61) subsequently used these resultant clusters to predict a range of post-assault outcomes, including PTSD symptoms, which were assessed at a one-year follow-up. The findings indicated a significant difference amongst three clusters in post-assault PTSD symptoms. “Alcohol-related assault” victims experienced lower number of PTSD symptoms than “high-violence assault” victims but higher number of symptoms than “moderate-severity assault” victims. However, the difference between “high-violence” and “alcohol-related assault” victims in the number of PTSD symptoms decreased over time, resulting in no significant difference 1 year later.

#### Cross-Sectional Studies of Acute Substance Intoxication

Jaffe et al. (70) assessed the role of acute alcohol intoxication (use and non-use of alcohol) shortly before sexual assault, as well as in relation to the level of acute alcohol intoxication at the time of the assault. This cross-sectional study showed that intoxication at the time of the assault was associated with a *greater* probability of reporting any PTSD symptoms even after controlling for the severity of coercion during the assault. Unlike Blayney et al. (62), they found a dose-dependent effect of acute alcohol intoxication on PTSD symptoms. Jaffe et al. (70) also showed that when controlling for coercion severity, participants who reported being “very intoxicated,” had significantly more severe PTSD symptoms than participants who reported lower levels of intoxication. In addition, there was a non-significant trend suggesting low and moderate levels of intoxication predicted lower PTSD severity when compared to non-intoxication. These dose-dependent effects of acute alcohol intoxication were particularly strong for re-experiencing PTSD symptoms.

Zinzow et al. (68) categorized sexual assault experiences into three different types: (a) “forcible rape” in which the perpetrator used force or threat of force; (b) “drug-or-alcohol-facilitated/incapacitated rape” in which victims were intoxicated and incapacitated via *voluntary or involuntary* consumption of drugs and/or alcohol during an adulthood sexual assault incident; (c) “combined type” rape which is defined as sexual assault experiences in which both force and incapacitation were used in the same incident. Lifetime and past 6-month PTSD outcomes of victims of “forcible rape,” “drug-or-alcohol-facilitated/incapacitated,” and “combined type” rape were compared with those of non-victims with no history of sexual assault. All types of sexual assault experiences were significantly related to the development of PTSD. However, victims of “combined type” rape exhibited the highest risk, followed by forcible rape and drug-or-alcohol-facilitated/incapacitated rape. Specifically, victims reporting “combined type” assaults were found to have over four times the likelihood of developing lifetime PTSD and six times the likelihood of developing PTSD over the past 6 months, compared to non-victims. Victims reporting “forcible rape” were more than twice as likely to develop lifetime PTSD and more than four times as likely to develop past 6-month PTSD as non-victims. Victims reporting “drug-or-alcohol-facilitated/incapacitated rape,” nonetheless, were more than twice as likely to develop lifetime and past 6-month PTSD as non-victims. This study, however, did not directly compare “drug-or-alcohol facilitated/incapacitated” rape with “forcible rape” or “combined type” rape.

In the studies by Zinzow et al. (67) and Zinzow et al. (66), sexual assault experiences were categorized differently to those in Zinzow et al. (68): (a) “forcible rape” in which the perpetrator used force or threat of force; (b) “incapacitated rape” in which the victim was intoxicated or impaired via *voluntary* intake of drugs or alcohol; and (c) “drug- or alcohol-facilitated rape” if the perpetrator deliberately attempted to produce incapacitation by administering drugs or alcohol to the victim so that they were intoxicated *involuntarily*. Both studies showed that forcible rape was associated with the highest risk of PTSD in comparison to incapacitated and drug- or alcohol-facilitated rape. Specifically, in Zinzow et al.'s (67) study, victims of various types of sexual assault experiences were compared with non-victims who had no history of sexual assault. The findings revealed that women who reported “forcible rape” were over 3 times as likely as non-victims to meet lifetime criteria for PTSD, even after controlling for other rape experiences and revictimization history. Victims who reported “drug- or alcohol-facilitated rape” were almost twice as likely as non-victims to meet lifetime criteria for PTSD. Victims who reported “incapacitated rape,” however, did not significantly differ from non-victims in terms of developing lifetime PTSD. In addition, the risk of developing lifetime PTSD was significantly higher for victims reporting “forcible rape” in comparison to victims reporting “incapacitated rape.” The odds ratio for victims reporting “drug- or alcohol-facilitated rape” did not differ from those reporting “forcible rape” or “incapacitated rape.” The odds ratio, nonetheless, was significantly higher for “forcible rape,” in comparison to “incapacitated rape.”

Zinzow et al. (66) indicated that all three types of sexual assault were associated with PTSD, although victims reporting “forcible rape” were four times as likely to meet lifetime criteria for PTSD as victims without a history of “forcible rape.” Victims reporting “drug- or alcohol-facilitated rape” had more than three times the likelihood of meeting lifetime PTSD criteria and those reporting “incapacitated rape” were approximately twice as likely to develop PTSD as victims without such experiences.

Taking a slightly different approach to classification of rape experiences, Brown et al. (64) compared “forcible rape” with both “incapacitated rape” and verbally coerced sexual assault experiences. “Verbal coercion” was defined as victims responding to unwanted sexual experiences because they were “overwhelmed by someone's continual arguments and pressure” or because someone used a position of authority to coerce them. They defined “incapacitated rape” differently from the studies cited above as victims reporting that they had unwanted sex because they were “incapable of giving consent or resisting due to alcohol or drugs.” Two studies were reported by Brown et al. (64). Study 1 assessed the most severe unwanted sexual assault experiences in a college sample and found all three groups (forcible, incapacitated, and verbally coerced) differed significantly from one another on the number of PTSD symptoms. Consistent with the prior studies, “forcible rape” victims reported the highest number of PTSD symptom, followed by “incapacitated rape” and “verbal coercion” victims, after controlling for the number of unwanted sexual assault experiences. Study 2 investigated the most recent experiences of a more diverse community sample. Victims who reported experiencing multiple methods of coercion were categorized according to the most coercive method (e.g., victims experiencing both verbal coercion and force were classified as “forcible rape” victims). Findings showed that victims of “verbal coercion” had significantly fewer number of PTSD symptoms than did “forcible rape victims.” “Incapacitated rape” victims reported an intermediate number of PTSD symptoms that was not significantly different from that of either of the other groups.

Littleton et al. (65) investigated sexual assault experiences of “impaired,” “incapacitated” and “non-impaired victims.” To be classified as “impaired” or “incapacitated,” victims needed to report impairment due at least in part to substance use. Victims who recounted being unconscious (due to acute substance-induced intoxication) during the assault were classified as “incapacitated,” while those reporting less severe forms of impairment (e.g., asleep, having trouble walking) were classified as “impaired.” To be classified as “non-impaired,” victims had to have experienced sexual assault that was not preceded by any type of drug/alcohol-induced impairment or incapacitation. No significant difference in the number of PTSD symptoms was found amongst these groups. It should be noted that the lack of significant difference in this study may be related to its methodological weaknesses (rated as medium in quality and relevance rating) and relatively smaller sample size than other studies with the similar design.

Masters et al. (69), similar to Peter-Hagene and Ullman (61)'s study using a data-driven approach, identified subgroups of sexual assault victims based on multiple characteristics of their assault experiences using latent class analysis. The subgroup structure was subsequently validated in a second cohort recruited in an identical manner to the first. They identified three substantially different subgroups: (a) “contact or attempted assault” (victims of attempted rape or contact sexual assault such as sexual harassment with no act of victimization by penetration); (b) “incapacitated assault” (victims of rape reporting prior incapacitation by a substance); and (c) “forceful severe assault” (victims of completed rape who were not incapacitated reporting force as the predominant characteristic of the assault). The results indicated that in terms of post-assault psychological distress, women in the “forceful severe assault” subgroup, compared with the other two subgroups, had significantly higher number of PTSD symptoms (e.g., intrusive thoughts and defensive avoidance) over the past 6 months. Moreover, victims in this group also reported more episodes of binge drinking in the past year than did victims in the “incapacitated assault” group.

#### Longitudinal Studies of Chronic Pre-assault Problematic Substance Use

Kaysen et al. (21) examined longitudinally the effects of AUD, self-reported maximum number of drinks and alcohol-related negative consequences for 30 days prior to the assault on different clusters of PTSD symptoms. The victims had experienced sexual or physical assault and were assessed within 5 weeks of the assault and again at 3 and 6 months post-assault. Alcohol-related negative consequences were measured at each time point to allow the researchers to examine a) severity of baseline drinking consequences experienced during the 30 days prior to the assault and b) changes in negative consequences from baseline to 3 months and from baseline to 6 months. Similar to their previous study (41), these authors used independent samples of victims with and without drinking problems to test the effects of chronic pre-assault problematic substance use on PTSD symptoms. Findings suggested that AUD and alcohol-related negative consequences (e.g., hangover, loss of interest in activities and hobbies due to drinking) were associated with significantly lower reports of PTSD symptoms immediately post-trauma exposure, even after controlling for demographics, trauma and psychological variables. There was no significant decrease in PTSD symptoms over time amongst victims with pre-existing AUD. Likewise; changes in alcohol-related negative consequences over time did not significantly interact with changes in PTSD symptoms. For those reporting high levels of alcohol-related negative consequences during the 30 days pre-assault, their PTSD symptoms did not decrease significantly over time. It was also shown that no individual cluster of PTSD symptoms accounted for this association, and the association of PTSD symptoms with maximum number of drinks was not significant. Similar to Kaysen et al.'s (41), this study had a small sample size and did not report the number of physical or sexual assault victims, respectively, nor differentiate between them in their PTSD symptoms.

In addition, Kaysen et al. (45) longitudinally assessed victims' PTSD symptoms 2–4 weeks and 3 months after the experience of sexual or physical assault. They used independent samples of victims with and without pre-assault AUDs and compared their post-assault PTSD symptoms. As a result, they reported that victims with pre-assault AUDs showed significantly more severe intrusion and avoidance, but not hyperarousal symptoms, than those without pre-assault AUDs. They also found that victims who had pre-assault AUDs continued to have more severe PTSD symptoms over time than victims without such histories, thus experiencing less symptom improvement over time. This interactive effect between pre-assault AUDs and time was only significant for hyperarousal symptoms, not for avoidance or intrusion symptoms, suggesting that only hyperarousal symptoms improved over time in victims with pre-assault AUDs. Similarly, this study did not report the number of physical or sexual assault victims, respectively, nor differentiate between them in their PTSD symptoms.

#### Mediators Between Acute Alcohol Intoxication and PTSD: Self-Blame

Three longitudinal studies examined the mediating role of post-assault self-blame on the relationship between acute alcohol intoxication and PTSD symptoms. Mediating variables were not examined for any other drug types in the reviewed papers. Peter-Hagene and Ullman (61) measured self-blame using the Self-Blame Attribution Questionnaire (96), which is composed of two 5-item subscales assessing both characterological and behavioral self-blame. Characterological self-blame attributions are dispositional beliefs about one's own character, reflecting beliefs that the assault was a result of who the victim was “as a person” or that the assault was somehow deserved. Behavioral self-blame attributions, on the other hand, are situation-specific beliefs about one's actions (e.g., drinking excessively) before the assault. This study showed that although assault characteristics predicted both behavioral and characterological self-blame, “high-violence” and “alcohol-related assault” types were related to increased PTSD only via characterological self-blame as a mediator. Overall, characterological self-blame was positively related to PTSD, and it mediated the difference between “high-violence” and “moderate sexual-severity assaults.”

In a similar vein, Peter-Hagene and Ullman (63) found that victims who were intoxicated as a result of pre-assault drinking tended to report more behavioral and characterological self-blame than those who were not. Although the effect of drinking on characterological self-blame was less strong than its effect on behavioral self-blame, it was more consistent over time and was maintained over time. The effect of behavioral self-blame, however, has been shown to diminish over time. Although acute alcohol intoxication was associated with fewer PTSD symptoms, the overall findings suggested a positive indirect effect of acute alcohol intoxication on PTSD via characterological self-blame. Intoxicated victims with characterological self-blame reported increased PTSD symptoms.

Blayney et al. (62) reported findings that were inconsistent with those of Peter-Hagene and Ullman's (61, 63) studies. Blayney et al. (62) examined post-assault cognitions on three scales: (a) self (global evaluations of the self); (b) world (general evaluations of others/the state of the world and one's place in it); and (c) self-blame (beliefs that one is responsible for the assault, e.g., “the event happened because of the way that I acted”). All three scales were tested as potential mediators for the association between acute alcohol intoxication and PTSD symptoms in relation to both cumulative sexual assault experiences since the start of college and the most recent experience during the college years. Results indicated a lack of significant indirect effect of any of these cognition types on the relationship between acute alcohol intoxication at the time of the assault and PTSD symptoms.

#### Mediators Between Acute Alcohol Intoxication and PTSD: Social Reactions

One longitudinal study examined the mediating role of post-assault social reactions in relation to sexual assault in the context of alcohol intoxication. Peter-Hagene and Ullman (61) used the Social Reaction Questionnaire [SRQ; (97)] to measure how often victims received positive (e.g., emotional support, tangible support) and/or negative (e.g., controlling, blaming, egocentric responses, distracting the victim, or treating the victim differently) social reactions since the assault on a rating scale ranging from 0 (*never*) to 4 (*always*). This questionnaire further separated negative social reactions into “acknowledgment-without-support” (i.e., acknowledging the assault happened, but failing to give adequate support; misplaced efforts to control the victim's decisions) and “turning-against” reactions (i.e., blaming the victim, not believing her story) based on confirmatory factor analyses (98). The findings indicated that “high-violence” and “alcohol-related assault” types were specifically related to increased PTSD via “turning-against” social reactions. “Turning-against” social reactions also mediated the difference in PTSD symptoms between “high-violence” and “alcohol-related” vs. “moderate-sexual-severity assaults.”

## Discussion

### Summary of Main Findings

The purpose of this review was to provide an overview of the effects of acute substance intoxication as well as chronic pre-assault problematic substance use on the development of PTSD symptoms in the context of sexual assault. In total, seven studies showed initial lower levels of PTSD symptoms in intoxicated victims compared to non-intoxicated victims (41, 61, 63, 66–69). Two of these studies further showed a more chronic course of PTSD symptoms with less improvement over time in intoxicated than unintoxicated victims (41, 61). One study indicated a dose-dependent effect of acute substance intoxication, showing its positive association with PTSD severity only at high levels of intoxication (70). Jaffe et al. (70) and Kaysen et al. (41) showed that the effects of acute substance intoxication were particularly strong for re-experiencing PTSD symptoms such as intrusive memories. Three studies, however, found no evidence of effects of acute substance intoxication on any of PTSD symptoms (62, 64, 65).

In addition, two studies showed a more chronic course of PTSD in victims with chronic pre-assault problematic substance use, such as pre-assault AUDs, one of which showed initial lower levels of PTSD symptoms (45), whereas the other showed initial higher levels (21). Two studies identified characterological self-blame as a significant mediator of the effect of acute substance intoxication on PTSD (61, 63), and one of them also suggested negative post-assault social reactions as a significant mediator (61). One study, however, failed to find any mediator (62).

Based on the results from the current review, it appears that overall, acute substance intoxication is associated with initially decreased PTSD symptoms but a more chronic course of residual symptoms. These results are in line with the mutual maintenance model that although substances help reduce PTSD symptoms temporarily, they may simultaneously impede natural recovery from PTSD in the long run. The initially decreased PTSD symptoms may be because acute substance administration can dampen stress responses and impair acquisition of fear memories (99), which may in turn result in lower perceived severity of the assault and less posttraumatic distress. In addition, the effects on PTSD symptoms may be attributed to the impact of acute substance intoxication on memory and extinction learning. For instance, research suggested that alcohol may cause retrograde facilitation and anterograde impairment for emotional materials, such that it may facilitate memory for the events occurring prior to, but impair memory for the events after its administration, which in this case is the memory for the incident of sexual assault. Therefore, information about sexual assault might not be well-recalled after alcohol consumption, resulting in less psychological distress and an initial decrease in PTSD symptoms [for review, see (100)].

Furthermore, both human and animal studies showed that extinction learning under alcohol is slower, weaker and less context-specific (99, 101), suggesting one potential mechanism for persistent distress and chronic course of PTSD symptoms following sexual assault during alcohol intoxication. However, it should be noted that it is unclear whether effects seen with alcohol can generalize to other drugs, such as benzodiazepines or gamma-hydroxybutyrate (GHB), which have similar neuropsychological, pharmacological and subjective profiles to alcohol, and have been implicated in drug facilitated rape [i.e., “date rape”; (102)].

However, the particularly strong effect of acute substance intoxication on re-experiencing and intrusive memories, cardinal symptoms of PTSD, may be explained by the dual representation theory [DRT; (103)]. According to DRT, memory for an event is supported by contextual and sensation-based memory systems. Contextual memory representations (C-reps) are the basis for narrative memory, can be voluntarily retrieved, and are contextually bound. Sensory memory representations (S-reps) include low-level, sensation-based information pertaining to sensory and affective experiences. Typical memory encoding involves interconnected and equally salient C-reps and S-reps, whereas pathological encoding may occur during traumatic events, resulting in salient and enduring S-reps that are disconnected from corresponding C-reps without contextualizing sensory memories (103). As a result, the reactivation of S-reps (e.g., through reminders) can trigger perceptual re-experiencing of the event without information regarding the encoding context (e.g., intrusive memories and flashbacks). Research has found that substance intoxication, such as in the case of alcohol, may selectively impair contextual memories (104), so that intoxication at the time of sexual assault may intensify re-experiencing and intrusion symptoms by further increasing the disconnection between C-reps and S-reps. In turn, more frequent re-experiencing symptoms may foster a sense that the world is unsafe, potentially increasing hyperarousal or avoidance symptoms (70), potentially leading to PTSD.

In addition, research evidence suggests that substances, such as alcohol and benzodiazepines (105, 106), can lead to disturbances in rapid eye moment (REM) sleep, which, in turn, can suppress memory consolidation and result in a long-term impact on PTSD symptoms (107). Insufficient memory consolidation may lead the traumatic memory trace to stay primarily located in subcortical and primary perceptual areas (S-reps), leaving it tightly coupled to its autonomic and perceptual markers, without appropriately integrating in autobiographical, cortical memory networks (C-reps). Exposure to a trauma trigger subsequently results in the involuntary retrieval of traumatic memory that is not contextualized and that is fragmented in time (i.e., intrusive memories), consisting of primary sensory information (images, smell, sounds) that is linked to physiological fear symptoms (103). These explanations support the susceptibility hypotheses that substance users may be more susceptible to PTSD following a traumatic event due to impaired psychological processes (e.g., memory) resulting from extensive substance consumption.

The dose-dependent effect of acute alcohol intoxication shown in Jaffe et al.'s (70) study might be related to the effect of amnesia resulting from high levels of acute intoxication. A number of research studies show that the consumption of high-level amnesic substances can sometimes result in amnesia for the trauma, especially in some cases of involuntary intoxication. Due to the lack of recall of the traumatic experience, victims with amnesia tend to wonder about what has happened and imagine the worst-case scenario, which, in turn, can lead to negative interpretations of the assault and hence various anxiety and PTSD symptoms, including fear, avoidance, nightmares and intrusive thoughts (108). In addition, despite alcohol-related memory impairment at high levels of intoxication, victims are likely to retain memory from before and after the trauma (109) that also contributed to the development of intrusive memories. However, Jaffe et al.'s findings differ from those of Bisby et al. (110). They conducted studies examining the effects of acute alcohol intoxication on intrusive memories in an experimental human model of intrusive memory formation involving video scenarios of road traffic accidents. They found a different dose-dependent effect of alcohol on intrusive memories, with a low dose increasing memory intrusions and a high dose decreasing intrusive symptoms. Differences in study methodologies and samples likely contributed to these differing results. For instance, it is likely that the levels of intoxication achieved in the controlled laboratory environment with healthy volunteers (e.g., with no or low levels of AUD symptoms) are lower than those that would be experienced personally in a real-world setting.

Several reviewed studies suggested significant mediating factors, providing some evidence for the third variable model. They showed that characterological, but not behavioral, self-blame, mediated the effects of acute substance intoxication, contributing to the chronic course of PTSD symptoms over time. Previous studies have reported similar findings that characterological self-blame is related to poorer recovery outcomes (111). Although the use of substances is a specific behavior, its links to characterological self-blame might be driven by strong societal stereotypes about the use of alcohol and drugs, especially among women who can be viewed as “loose,” or “bad,” and deserving punishment (112, 113). As a result, individuals tend to blame themselves for the assault and identify with these societal stereotypes if they had been drinking or taking drugs, resulting in characterological self-blame, which is more strongly related to PTSD over time.

Post-assault social reactions also appear to play a role in the chronicity of PTSD symptoms, although evidence for this is based only on one single study. Sexual assault victims with acute substance intoxication tended to experience more blame and disbelief from others and hence receive more negative social support than victims without intoxication (114). There is ample evidence that negative social reactions contribute to PTSD symptoms, although positive social support does not appear to protect against PTSD (115–117). In addition, due to aversive social responses, these victims are less likely to seek help or talk about the assault with others, leading to more maladaptive individual and social coping strategies including avoidance, denial and social withdrawal. These, in turn may hinder recovery from PTSD symptoms (98, 118).

Due to the limited number of studies and inconsistent findings (21, 45), it is difficult to draw any conclusion regarding the effects of chronic pre-assault problematic substance use on PTSD symptoms. The inconsistent findings may be attributed to the different sample sizes (both relatively small) and the course and onset of pre-assault substance problems. The time for the follow-up PTSD assessments also varies between studies, and PTSD symptoms were examined either in clusters or as a whole, which may lead to differential outcomes. In addition, two studies examining the effects of chronic pre-assault problematic substance use included both physical and sexual assault victims, so the outcomes may not be generalizable to studies with sexual assault victims only. Lastly, these two studies both investigated AUDs, possibly leading to different results from the effects of other drugs.

### Limitations

Although the review was designed to include both male and female sexual assault victims, there was only one study comprising both genders [with only 19% male college victims in a total sample of 116; (62)]. Previous research suggested gender differences in that women appear more vulnerable to alcohol-related consequences at lower levels of alcohol exposure than men. In general, women tend to have more fatty tissue than men and given the relative solubility of alcohol in fat and water, this result in greater blood alcohol levels for the same amount (g/kg) of consumed alcohol in women relative to men. In addition, women usually have lower gastric dehydrogenase activity in the stomach to metabolize alcohol, so that after an equivalent dose of alcohol, women have higher blood ethanol levels than men and hence greater vulnerability to the consequences of drinking alcohol (119). Results from alcohol research and clinical studies highlight that gender differences in alcohol impairment may be due also to the action of sex hormones (especially estrogen), which can modulate alcohol effects and alcohol itself may modulate hormonal status. For instance, estrogen administration repeatedly increased hepatic dehydrogenase activity (120). Therefore, the findings in this current review, which mostly consisted of studies in female victims, may not generalize to male victims.

In addition, most reviewed studies reported the impact of pre-assault alcohol consumption, whereas there was little extant information on the impact of other types of substances, limiting the generalizability of these findings. Research also highlighted that the vast majority of victims who use drugs also consume alcohol (121), so the co-occurrence may bring challenges in separating the outcomes. In addition, the reviewed studies did not report the type of drugs involved in the assault. Research studies show that stimulant drugs (e.g., nicotine, cocaine, methamphetamine) and depressant drugs (e.g., heroin, GHB, benzodiazepine) affect the body and brain functions differently [e.g., (100)] and may result in different effects on the development of PTSD symptoms.

The inclusion of diverse designs (cross-sectional and longitudinal) might be considered a limitation of the current review. However, this was necessary to obtain a comprehensive understanding of the impact of pre-assault substance consumption on PTSD in a qualitative systematic review. This review included community and college samples and samples from specific agencies with mostly large samples over multiple time points. Despite this breadth, there remained some variations in methodological strengths across the reviewed studies. In addition, unlike a meta-analysis, this review places the same value on studies with small sample sizes as those with extensive sample sizes, and hence do not take into consideration of the impact of sample size on the validity of outcomes. For this current review, therefore, it is important that methodologically weaker studies, including those with lower quality and relevance ratings, smaller sample sizes and shorter follow-up periods, should be given less weight.

Similarly, there were variations in the use of different measures for assessing PTSD symptoms and sexual assault experiences, resulting in a lack of consistency in variable definitions. In addition, some studies took baseline measures shortly after the assault, whereas others collected the data long after the assault had occurred. This may lead to problems in comparing results across studies due to potential confounding variables.

As shown in Jaffe et al.'s (70) study, there may be a dose-dependent effect of acute substance intoxication. The levels of acute substance intoxication were not reported in most of the reviewed studies, and it was possible that they varied across studies, contributing to inconsistent findings that for instance, low levels of substances would impact PTSD symptoms differently from high levels. Furthermore, it is reasonable to hypothesize that the effects of chronic pre-assault problematic substance use may also be dose-dependent, possibly leading to different degrees of PTSD symptoms depending on the severity of pre-assault substance problems. Therefore, further studies need to be conducted to explore this hypothesis.

The number of studies reviewed here was relatively low, with 13 in total. In addition, there were only two studies investigating the effect of chronic pre-assault problematic substance use on PTSD symptoms. As a result, it is difficult to make generalizable conclusions based on these limited findings. It should also be noted that no mediator between chronic pre-assault problematic substance use and PTSD symptoms, and only two mediators between acute substance intoxication and PTSD symptoms have been studied in the extant literature. Hence, there may potentially be other mediators that need to be explored further.

### Conclusions and Future Directions

The findings reported in this review suggest that lower initial PTSD symptoms following trauma exposure amongst substance consumers may not necessarily indicate reduced risk for PTSD over time. Given that early interventions for victims of sexual assault may not be offered to those who initially present with lower PTSD symptoms, it is possible that these particular individuals may be less likely to receive such early interventions for PTSD (122). Moreover, because of shame, stigma and negative social reactions, including the tendency to “blame the victim,” even victims with severe PTSD symptoms may not receive early help as a result of their failure to seek it out. Therefore, the findings from this review suggest a need for routinely assessing both pre-assault and post-assault substance consumption (123) in order to detect l victims who might develop chronic PTSD and provide appropriate early interventions. In addition, previous research supports providing a brief PTSD intervention for trauma-exposed individuals who are also experiencing difficulties with alcohol in order to facilitate natural recovery from drinking problems. Conversely, reducing the degree of problems associated with alcohol use could, in turn, encourage PTSD recovery over time (124). Therefore, interventions addressing one of the problems in an acute trauma-exposed sample could be helpful in alleviating the other.

Furthermore, the findings of this review help identify mediators for PTSD development following sexual assaults (i.e., characterological self-blame and negative social reaction). This is key in appropriately targeting the focus of interventions and hence developing effective prevention programmes for the victims (125). Specifically, our findings suggested that early interventions should target and focus on areas of self-blame and the development of social support to help victims recover from the trauma effectively.

This review also highlights some gaps in this field of research. Little is known about the impact of substances other than alcohol on PTSD development amongst victims of sexual assault. In addition, very limited research has been conducted with male victims of sexual assault. Therefore, future research should be carried out in these areas. Additionally, since all studies were conducted in the US, this clearly limits generalizability to low- and middle-income countries. Given differences in attitudes toward sexual behavior and the use of substances between the US and, for example, European countries (126), the results might not be generalizable to other high-income countries.

Longitudinal studies with prolonged follow-up periods would also be helpful in understanding the development of post-assault PTSD symptoms and investigating the outcomes of different levels of acute substance intoxication and chronic pre-assault problematic substance use. More laboratory-based studies should aim to establish the causal relationship between pre-assault substance consumption and PTSD. Lastly, as PTSD and SUDs have been shown to be closely associated, it would be invaluable to design and evaluate intervention programmes that address these problems concurrently within the trauma-exposed population.

## Author Contributions

AG, SK, and HC contributed to the conception and design of the review. AG organized the database, reviewed the papers, and wrote the first draft of the manuscript. All authors contributed to manuscript revision, read, and approved the submitted version.

### Conflict of Interest Statement

The authors declare that the research was conducted in the absence of any commercial or financial relationships that could be construed as a potential conflict of interest.
