# Supplementary material for: Post-traumatic Stress Disorder in Victims of Sexual Assault With Pre-assault Substance Consumption: A Systematic Review
Source: Front Psychiatry. 2019 Mar 13;10:92. doi: 10.3389/fpsyt.2019.00092 (PMC6424881; doi:10.3389/fpsyt.2019.00092)
Supplement: Supplementary file 1 [file Table_1.DOCX]

Supplementary Material

Post-Traumatic Stress Disorder in Victims of Sexual Assault with Pre-assault Substance Consumption: A Systematic Review

An Tong Gong^1^, Sunjeev K. Kamboj^1^, H. Valerie Curran*

^1^ Clinical Psychopharmacology Unit, Research Department of Clinical, Educational and Health Psychology, University College London (UCL), London, UK

*** Correspondence:** Helen Valerie Curran
Address: Clinical Psychopharmacology Unit, Research Department of Clinical, Educational and Health Psychology, University College London (UCL), Gower Street, London, WC1E 6BT, UK
Tel: 020 7679 1898
Fax: 020 7916 1989

Email: v.curran@ucl.ac.uk

## Quality and relevant assessment scales

**Adapted from Newcastle-Ottawa Quality Assessment Scale**

**(longitudinal/cohort study)**

*Italics represent changes from original assessment scale*

Note: A study can be awarded a maximum of one star for each numbered item within the Selection and Outcome categories. A maximum of two stars can be given for Comparability.

**Selection (Maximum 4 stars)**

1) Representativeness of the exposed cohort

a) truly representative of the average *victims of SA* in the community *

b) somewhat representative of the average *victims of SA* in the community *

c) selected group of users *(e.g. using specialist service or with a particular need)*

d) no description of the derivation of the cohort

2) Selection of the non-exposed cohort

a) drawn from the same community as the exposed cohort *

b) drawn from a different source

c) no description of the derivation of the non-exposed cohort

3) Ascertainment of exposure

a) secure record *(e.g. police record or report)* *

b) structured interview *

c) written self-report

d) no description

4) Demonstration that outcome of interest was not present at start of study

a) yes *

b) no

**Comparability (Maximum 2 stars)**

1) Comparability of cohorts on the basis of the design or analysis

a) study controls for *baseline mental health condition (except for SUDs)* *

b) study controls for *demographics (e.g. age, race/ethnicity, sexuality, education background, marital status, employment status, income level etc.)* *

**Outcome (Maximum 4 stars)**

1) Assessment of outcome

a) independent blind assessment *

b) record linkage *

c) self-report

d) no description

2) Was follow-up long enough for outcomes to occur

a) yes *(follow up >/= 6 months)* *

b) no *(follow up < 6 months)*

3) Adequacy of follow up of cohorts

a) complete follow up - all subjects accounted for *

b) subjects lost to follow up unlikely to introduce bias - small number lost - *</= 20%* follow up, or description provided of those lost *

c) follow up rate *< 80%* and no description of those lost

d) no statement

**Adapted from Newcastle-Ottawa Quality Assessment Scale**

**(adapted for cross-sectional studies)**

*Italics represent changes from original assessment scale*

Note: A study can be awarded a maximum of one star for each numbered item within the Selection and Outcome categories. A maximum of two stars can be given for Comparability.

**Selection: (Maximum 4 stars)**

1) Representativeness of the sample

a) truly representative of the average *victims of SA* in the community *

b) somewhat representative of the average *victims of SA* in the community *

c) selected group of users *(e.g. using specialist service or with a particular need)*

d) no description of the derivation of the cohort

1. Sample size
2. Justified and satisfactory *
3. Not justified

3) Non-respondents

a) Comparability between respondents‘ and non-respondents’ characteristics is established, and the response rate is satisfactory. *

b) The response rate is unsatisfactory, or the comparability between respondents and non-respondents is unsatisfactory.

c) No description of the response rate or the characteristics of the responders and the non-responders.

4) Ascertainment of the exposure (risk factor):

a) Validated measurement tool *

b) Non-validated measurement tool, but the tool is available or described

c) No description of the measurement tool

**Comparability (Maximum 2 stars)**

1) The subjects in different outcome groups are comparable, based on the study design or analysis. Confounding factors are controlled.

a) study controls for *baseline mental health condition (except for SUDs)* *

b) study controls for *demographics (e.g. age, race/ethnicity, sexuality, education background, marital status, employment status, income level etc.)* *

**Outcome:(Maximum 2 stars)**

1) Assessment of the outcome:

a) Independent blind assessment *

b) Record linkage *

c) Self-report

d) No description

2) Statistical test:

a) The statistical test used to analyse the data is clearly described and appropriate, and the measurement of the association is presented, including confidence intervals and the probability level (p value). *

b) The statistical test is not appropriate, not described or incomplete.
